# Epidemiological characteristics of respiratory viruses in hospitalized children during the COVID-19 pandemic in southwestern China

**DOI:** 10.3389/fcimb.2023.1142199

**Published:** 2023-04-04

**Authors:** Lin Zhu, Tingting Luo, Yining Yuan, Shu Yang, Chao Niu, Ting Gong, Xueer Wang, Xiaohong Xie, Jian Luo, Enmei Liu, Zhou Fu, Daiyin Tian

**Affiliations:** ^1^ Ministry of Education Key Laboratory of Child Development and Disorders, Chongqing Key Laboratory of Pediatrics, Department of Respiratory Medicine, National Clinical Research Center for Child Health and Disorders, Children’s Hospital of Chongqing Medical University, Chongqing, China; ^2^ School of Public Health, Chengdu Medical College, Chengdu, China; ^3^ College of Intelligent Medicine, Chengdu University of Traditional Chinese Medicine, Chengdu, China

**Keywords:** COVID-19, SARS-CoV-2, nonpharmaceutical interventions, epidemiology, pediatrics, respiratory virus

## Abstract

**Background:**

Multinational studies have reported that the implementation of nonpharmaceutical interventions (NPIs) to control severe acute respiratory syndrome coronavirus 2 (SARS-CoV-2) transmission coincided with the decline of other respiratory viruses, such as influenza viruses and respiratory syncytial virus.

**Objective:**

To investigate the prevalence of common respiratory viruses during the coronavirus disease 2019 (COVID-19) pandemic.

**Methods:**

Respiratory specimens of children with lower respiratory tract infections (LRTIs) hospitalized at the Children’s Hospital of Chongqing Medical University from January 1, 2018 to December 31, 2021 were collected. Seven common pathogens, including respiratory syncytial virus (RSV), adenovirus (ADV), influenza virus A and B (Flu A, Flu B), and parainfluenza virus types 1–3 (PIV1–3), were detected by a multiplex direct immunofluorescence assay (DFA). Demographic data and laboratory test results were analyzed.

**Results:**

1) A total of 31,113 children with LRTIs were enrolled, including 8141 in 2018, 8681 in 2019, 6252 in 2020, and 8059 in 2021.The overall detection rates decreased in 2020 and 2021 (*P* < 0.001). The detection rates of RSV, ADV, Flu A, PIV-1, and PIV-3 decreased when NPIs were active from February to August 2020, with Flu A decreasing most predominantly, from 2.7% to 0.3% (*P* < 0.05). The detection rates of RSV and PIV-1 resurged and even surpassed the historical level of 2018–2019, while Flu A continued decreasing when NPIs were lifted (*P* < 0.05). 2) Seasonal patterns of Flu A completely disappeared in 2020 and 2021. The Flu B epidemic was observed until October 2021 after a long period of low detection in 2020. RSV decreased sharply after January 2020 and stayed in a nearly dormant state during the next seven months. Nevertheless, the detection rates of RSV were abnormally higher than 10% in the summer of 2021. PIV-3 decreased significantly after the COVID-19 pandemic; however, it atypically surged from August to November 2020.

**Conclusion:**

The NPIs implemented during the COVID-19 pandemic affected the prevalence and seasonal patterns of certain viruses such as RSV, PIV-3, and influenza viruses. We recommend continuous surveillance of the epidemiological and evolutionary dynamics of multiple respiratory pathogens, especially when NPIs are no longer necessary.

## 1 Introduction

Coronavirus disease 2019 (COVID-19), caused by severe acute respiratory syndrome coronavirus 2 (SARS-CoV-2), has rapidly spread worldwide since December 2019 (Zhu et al., 2020). The impact of the COVID-19 pandemic on public health was catastrophic, with millions of hospitalizations and deaths. In response to the ongoing COVID-19 pandemic, the Chinese government undertook a series of stringent nonpharmaceutical interventions (NPIs) as soon as it outbroke at the end of 2019 in Wuhan, including SARS-CoV-2 detection, home quarantining, contact tracing, mask mandates, regular hand-sanitizing and hand-washing, transient closures of public places, and maintaining social distance. Substantial evidence has shown that such NPIs effectively limited the transmission of SARS-CoV-2 (Lai et al., 2020; Grote et al., 2021; León et al., 2021; Zhao et al., 2021). Moreover, it has also been recorded that in hospitals and private practices, the number of visits for airborne or fecal-oral diseases (common cold, bronchiolitis, seasonal influenza, acute otitis media, and gastroenteritis) decreased significantly during the NPIs implementation period (Pan et al., 2020; Sakamoto et al., 2020).

The positive collateral effect of NPIs in the short term is welcome, as it helps to reduce the additional overload of hospital wards and intensive care units. Two years after the outbreak of the COVID-19 pandemic, public health measures were gradually eased. Business resumed, schools and kindergartens fully reopened in September 2020, and the SARS-CoV-2 vaccination campaign began in 2021. Several studies have reported that strict NPIs affected the transmission and seasonal circulation patterns of respiratory viral infections (Agca et al., 2021; Li et al., 2021; Yeoh et al., 2021; Song et al., 2022), such as influenza viruses and respiratory syncytial virus, and some studies later reported atypical increases in certain viruses after normalization in 2021. There have been increasing concerns that the strict mitigation measures and their subsequent relaxation might have modified the epidemiology of respiratory viruses, which could cause more severe epidemics when NPIs are no longer necessary. In view of these concerns, mastering epidemiological characteristics of common respiratory viruses during the COVID-19 pandemic is vital and essential for regional disease prevention and control.

To investigate the prevalence of common respiratory viruses during the COVID-19 pandemic, we retrospectively analyzed the absolute number and the detection rates of common respiratory viruses in children hospitalized for lower respiratory tract infections (LRTIs) at Children’s Hospital of Chongqing Medical University from January 1, 2020 to December 31, 2021. For comparison, the corresponding data in the same periods of 2018 and 2019 were also utilized.

## 2 Methods

### 2.1 Study design

Patients with LRTIs between January 1, 2018 and December 31, 2021 admitted to the respiratory ward of Children’s Hospital of Chongqing Medical University were enrolled. The inclusion criteria were as follows: 1) children under the age of 18 years hospitalized for LRTIs; 2) available results of multiplex direct immunofluorescence assay (DFA) for respiratory pathogens. The diagnoses of LRTIs included bronchiolitis, bronchitis, and pneumonia. Patients were excluded if they had one of the following situations: 1) children with severe malformations (such as large atrial/ventricular septal defects, bronchopulmonary dysplasia, dextrocardia, and neuromuscular disease); 2) children who received diagnosis of malignant tumors or primary immunodeficiency disease, or who used immunosuppressive drugs during hospitalizations.

All of the enrolled children were divided into five age groups: under 12 months (0–12 m), 1 to 3 years (1–3 y), 3 to 6 years (3–6 y), 6 to 14 years (6–14 y), and more than 14 years (>14 y). The period between January 1, 2018 and December 31, 2019 was classified as before the COVID-19 pandemic, while the period between January 1, 2020 and December 31, 2021 was classified as during the COVID-19 pandemic. The time span was further subdivided into three phases according to the emergency response level in Chongqing: January 24, 2020 to March 24, 2020 (Phase I, level I–II emergency response), when massive NPIs remained active; March 25, 2020 to August 31, 2020 (Phase II, level III emergency response), when NPIs were gradually relaxed and schools started reopening; September 1, 2020 to December 31, 2021 (Phase III, normalization of the epidemic), when schools fully reopened and social activities resumed. The same phases were also defined based on the corresponding calendar intervals for 2018–2019.

### 2.2 Specimens collection and detection

Nasopharyngeal aspirate or bronchoalveolar lavage fluid specimens of the enrolled children were collected by trained nurses after admission and were transported immediately to the clinical laboratory center. Seven common pathogens included respiratory syncytial virus (RSV), adenovirus (ADV), influenza virus A (Flu A), influenza virus B (Flu B), and parainfluenza virus types 1–3 (PIV1–3). A multiplex direct immunofluorescence assay kit (Diagnostic Hybrids, Athens, Ohio, USA) was used for detection and was conducted by professional staff following standard operating procedures (Ye and Liu, 2022).

It should be noted that when a child was hospitalized for LRTIs multiple times (interval less than 1 week), laboratory test results collected for the first hospitalization were included in the analysis. Respiratory specimens of the excluded patients were not included when calculating hospitalization numbers or detection rates.

### 2.3 Data collection

Demographic data (name, gender, months of age, clinical diagnosis, sampling time, and length of stay) of the patients were obtained from the electronic medical records, and laboratory test results were extracted from the management information system (HIS) of Children’s Hospital of Chongqing Medical University. The study protocol was approved by the Ethics Committee of Children’s Hospital of Chongqing Medical University (File No. (2022)196). Written consent was waived because antigen detection of respiratory pathogens was standard and routine for all children admitted to the hospital for LRTIs during the implementation of the study.

### 2.4 Statistical analysis

The overall detection rates of seven common pathogens, including RSV, ADV, Flu A, Flu B, and PIV1–3, were compared between the periods before and during the COVID-19 pandemic. To determine seasonal patterns of viruses and age-specific differences in viral infections, the trends of virus detection rates over time and the comparison of age-specific detection rates associated with viruses in 2018–2021 were plotted by software R version 4.1.3.

Data extracted from January 1, 2018 to December 31, 2021 were analyzed using IBM SPSS version 25.0. Categorical variables were described as numbers and percentages (n, %), and continuous variables were described as the median and interquartile range (IQR) [M (P_25_, P_75_)]. The Kruskal–Wallis test were used for statistical analysis of continuous variables, and Fisher’s exact test or Pearson chi-square test were used for categorical variables. *Post-hoc* pairwise comparisons were performed with partitions of the χ^2^ method, and the adjusted test level was α’ = 0.0083. Other tests were based on a two-sided α with statistical significance defined as P < 0.05.

## 3 Results

### 3.1 Study population

A total of 31,133 children hospitalized with LRTIs were admitted in this study, including 8141 in 2018, 8681 in 2019, 6252 in 2020, and 8059 in 2021. In 2020, the number of hospitalizations for LRTIs decreased by 23.2% and 28.0% compared with 2018 and 2019, respectively. A total of 19,124 (61.4%) cases were male and 12,009 (38.6%) were female. The age of the enrolled children ranged from 17 days to 18 years, and the children’s median (IQR) age was 12.0 (4.8, 34.0) months (**Table 1**).

**Table 1:** Comparison of the characteristics and virus detection rate before and during the COVID-19 pandemic.

	Before the COVID-19 pandemic		During the COVID-19 pandemic		H/χ^2^	P
	2018, n=8141	2019, n=8681	2020, n=6252	2021, n=8059		
Characteristics						
Sex(male)	5016 (61.6)	5352 (61.7)	3909 (62.5)	4847 (60.1)	9.080	0.028
Age, m	9.6 (4.0,22.0)	13.0 (5.0,37.0)	11.2 (4.0,30.9)	14.5 (6.0,40.0)	471.229	<0.001
Length of stay, d	6.0 (4.0,7.0)	5.3 (4.0,7,0)	5.9 (4.0,7.8)	5.9 (4.0,7.7)	87.432	<0.001
Virus detection rate						
Overall detection rate	3026 (37.2)	3033 (34.9)	1580(25.3)	2388 (29.6)	283.698	<0.001
Mixed infections rate	107 (1.3)	199 (2.3)	58 (0.9)	108 (1.3)	53.423	<0.001
Monthly detection rate						
Jan	348 (49.5)	340 (48.4)	364 (46.8)	252 (36.8)	28.173	<0.001
Feb	307 (46.4)	283 (44.4)	82 (16.2)	186 (35.8)	134.947	<0.001
Mar	288 (39.7)	288 (40.2)	9 (2.1)	132 (20.8)	260.507	<0.001
Apr	252 (37.0)	265 (38.0)	12 (2.7)	139 (19.9)	229.567	<0.001
May	178 (26.3)	203 (28.2)	7 (1.9)	108 (15.6)	129.684	<0.001
Jun	163 (25.8)	213 (29.2)	8 (2.8)	167 (24.0)	82.639	<0.001
Jul	157 (24.9)	212 (27.8)	25 (6.0)	185 (30.6)	95.870	<0.001
Aug	161 (23.5)	173 (23.7)	70 (15.2)	162 (25.2)	18.052	<0.001
Sep	137 (20.8)	127 (16.8)	108 (19.3)	134 (20.3)	4.335	0.227
Oct	258 (36.9)	163 (22.0)	233 (36.8)	273 (34.2)	50.141	<0.001
Nov	393 (56.6)	337 (45.5)	307 (48.7)	308 (44.3)	26.048	<0.001
Dec	384 (55.3)	429 (57.2)	355 (48.6)	342 (46.7)	22.557	<0.001

### 3.2 Overall detection of respiratory viruses before and during the COVID-19 pandemic

In 10,027/31,133 (32.2%) respiratory specimens of the enrolled children, one or more viruses were detected: 3026/8141 (37.2%) in 2018, 3033/8681 (34.9%) in 2019, 1588/6252 (25.3%) in 2020, and 2388/8059 (29.6%) in 2021. The overall detection rates in 2020 and 2021 were significantly lower than in prior years (*P* < 0.001). In the present study, two or more viruses were detected in 472 respiratory aspirates (107 in 2018, 199 in 2019, 58 in 2020, and 108 in 2021). Mixed infection rates were significantly different over four years (χ^2^ = 53.423, *P* < 0.05), with the rate in 2020 being lower than that in 2019 (χ^2^ = 40.107, *P* < 0.001). Monthly detection rates declined sharply from February to August 2020. Less than 10% of respiratory aspirates of the hospitalized children with LRTIs were infected by one or more viruses from March to July 2020 (**Table 1**).

### 3.3 Prevalence of seven respiratory viruses before and during the COVID-19 pandemic

**Figure 1:**
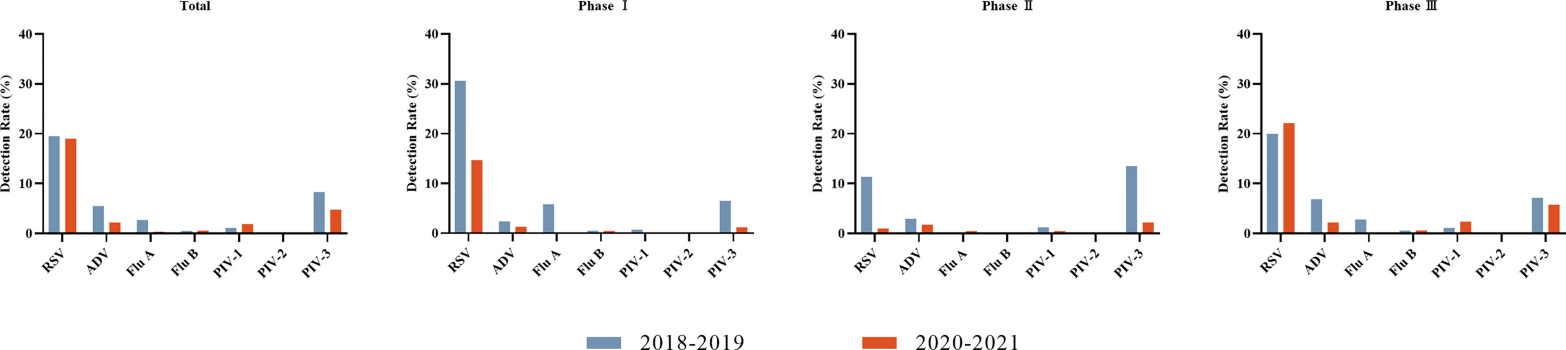
Detection rates of viruses before the COVID-19 pandemic (2018–2019) and during the COVID-19 pandemic (2020–2021) for each of three phases. Seven respiratory viruses were included: respiratory syncytial virus (RSV), adenovirus (ADV), influenza virus A and B (Flu A, Flu B), and parainfluenza virus types 1–3 (PIV1–3).

Overall, the detection rates of ADV, Flu A, and PIV-3 decreased during the COVID-19 pandemic (*P* < 0.05), while that of PIV-1 increased (*P* < 0.05). The detection rate of Flu A decreased most predominantly, from 2.7% to 0.3%. There were no significant differences in the detection rates of RSV, Flu B, and PIV-2 (*P* > 0.05). More specifically, dramatic reductions in the detection rates were seen for RSV, Flu A, and PIV-3 in Phase I, when NPIs were actively maintained (*P* < 0.05). The detection rates of RSV and PIV-3 continued decreasing despite partial relaxation of the mass NPIs in Phase II (*P* < 0.05). The detection rates of PIV-1 and ADV also lowered in Phase II (*P* < 0.05). Unlike Flu A, RSV and PIV-1 resurged in Phase III and even surpassed the historical level of 2018–2019 when NPIs were largely lifted in Chongqing, including full school reopening and resuming of social activities (*P* < 0.05). The detection rates of Flu B and PIV-2 were not significantly different in Phase I and Phase II compared with the pre-pandemic level (*P* > 0.05) **(** **Figure 1** and **Table 2** **)**.

**Table 2:** Detection rates of seven viruses before the COVID-19 pandemic (2018–2019) and during the COVID-19 pandemic (2020–2021) for each of three phases.

	Total				Phase I				Phase II				Phase III			
	2018-2019	2020-2021	χ^2^	P	2018-2019	2020-2021	χ^2^	P	2018-2019	2020-2021	χ^2^	P	2018-2019	2020-2021	χ^2^	P
RSV	3282(19.5)	2722(19.0)	1.192	0.275	427(30.6)	156(14.7)	84.44	<0.001	394(11.4)	21(1.0)	199.247	<0.001	2283(20.0)	2350(22.1)	15.461	<0.001
ADV	928(5.5)	315(2.2)	221.751	<0.001	33(2.4)	14(1.3)	3.524	0.060	102(2.9)	36(1.8)	7.590	0.006	787(6.9)	233(2.2)	274.522	<0.001
Flu A	460(2.7)	49(0.3)	275.141	<0.001	81(5.8)	1(0.1)	60.988	<0.001	2(0.1)	10(0.5)	–	0.001^#^	322(2.8)	18(0.2)	254.133	<0.001
Flu B	84(0.5)	84(0.6)	1.106	0.293	7(0.5)	5(0.5)	0.0120	0.913	0(0.0)	1(0.1)	–	0.373^#^	67(0.6)	67(0.6)	0.183	0.669
PIV-1	185(1.1)	271(1.9)	33.769	<0.001	10(0.7)	2(0.2)	3.466	0.063	43(1.2)	10(0.5)	7.763	0.005	128(1.1)	254(2.4)	52.340	<0.001
PIV-2	30(0.2)	14(0.1)	3.552	0.059	0(0.0)	0(0.0)	–	1^#^	1(0.0)	0(0.0)	–	1^#^	29(0.3)	11(0.1)	6.850	0.009
PIV-3	1396(8.3)	682(4.8)	154.957	<0.001	91(6.5)	13(1.2)	41.770	<0.001	469(13.5)	46(2.2)	195.226	<0.001	817(7.2)	613(5.8)	17.146	<0.001

Statistically significant changes were based on P-value < 0.05 by chi-square test or Fisher’s exact test^#^.

*Phase I: Jan 24^th^ to Mar 24^th^; Phase II: Mar 25^th^ to Aug 31^st^; Phase III Sep 1^st^ to Dec 31^st^ of the following year.

### 3.4 Seasonal patterns of respiratory viruses before and during the COVID-19 pandemic

The seasonal dynamics of common respiratory viruses was distorted during the COVID-19 pandemic. Detection of all seven viruses declined quickly after the outbreak on January 24, 2020, when a Level I public health emergency response was implemented in Chongqing. RSV usually peaks in winter and declines by early spring, as shown before the COVID-19 pandemic. However, the RSV detection rates decreased sharply from 35.99% in January 2020 to 12.82% in February 2020, and stayed in a nearly dormant state during the next seven months, without RSV-infected cases in April 2020. The monthly positive rate of RSV increased quickly to 16.43% in October 2020, peaked in winter, and then gradually decreased to 10.85% (75/691) in May 2021. However, the detection rates did not decrease continuously but increased again in June 2021 (12.23%, 85/695) and stayed abnormally high (over 10%) until September 2021.

The influenza virus is a pathogen with apparent seasonality patterns that shows high incidence in winter and spring (Wang et al., 2016). However, there was a sudden decline in Flu A and Flu B in January 2020, and nearly no influenza viruses were detected during the next several months of 2020. Seasonal patterns of Flu A completely disappeared in the winter of 2020 and 2021, but Flu B gradually rose until September 2021 and then peaked in October and November after a long period of very low detection.

PIV-3 circulated most frequently from late spring to summer in 2018 and 2019. However, no PIV-3 was detected from April to June 2020. However, the detection rates of PIV-3 atypically increased from August to November 2020, with a delay of about 5 months, and then fell before rising in June 2021. PIV-1 and PIV-2 were normally sporadically detected during the four years analyzed.

**Figure 2:**
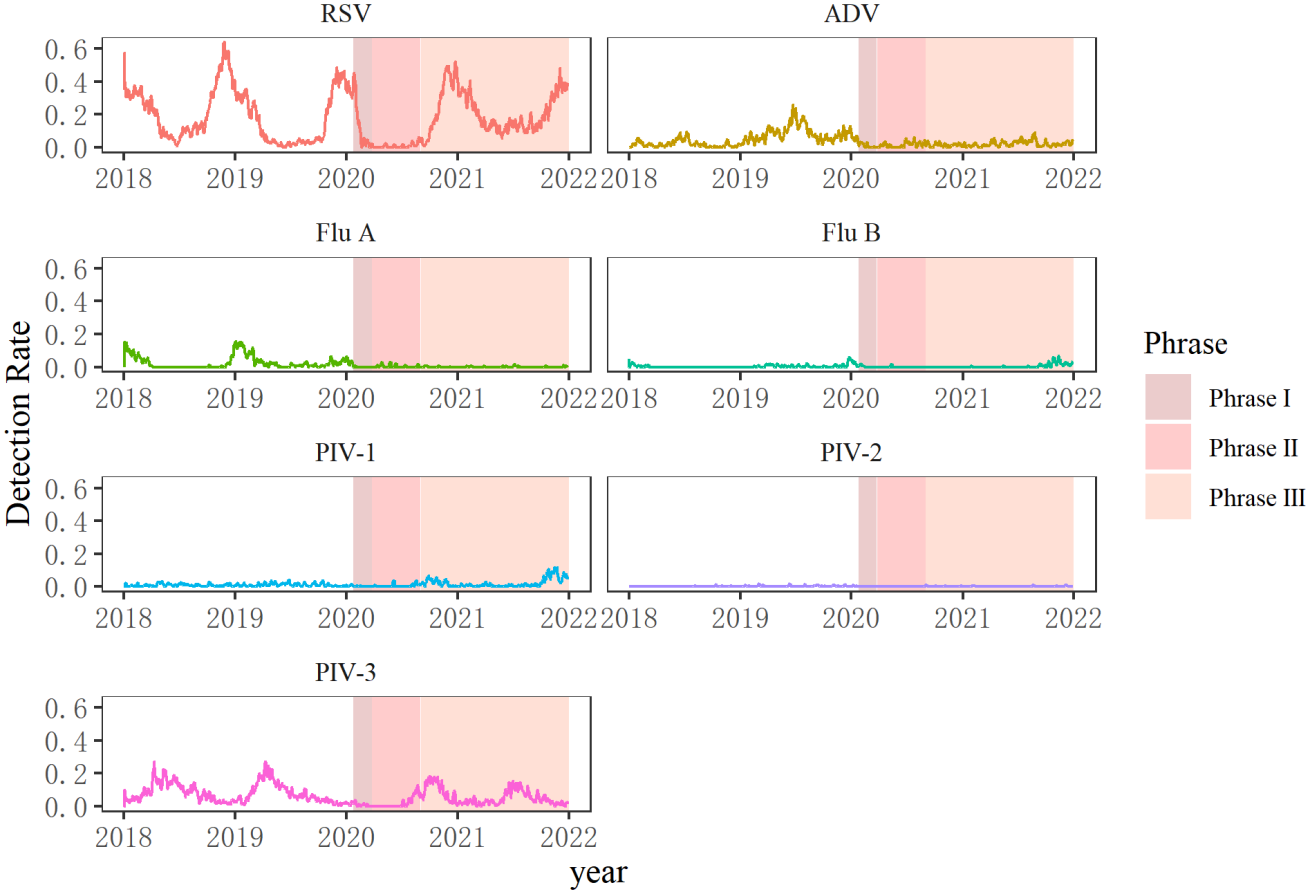
Seasonal patterns of viruses before the COVID-19 pandemic (2018–2019) and during the COVID-19 pandemic (2020–2021). Seven respiratory viruses were included: respiratory syncytial virus (RSV), adenovirus (ADV), influenza virus A and B (Flu A, Flu B), and parainfluenza virus types 1–3 (PIV1–3).

The detection rate of ADV in 2019 was extremely high all year round, peaking in summer. Infection caused by ADV can occur throughout the year; it mainly occurs in winter and spring in northern China, and is common in spring and summer in southern China (Ji et al., 2021). In 2019, the detection rate of ADV was high all year round in Chongqing. ADV peaked between June and August in 2020 and 2021, similar to 2018 and 2019 (**Figure 2**; **Supplementary Figure**).

### 3.5 Comparison of the detection rates of respiratory viruses among different age groups before and during the COVID-19 pandemic

**Figure 3:**
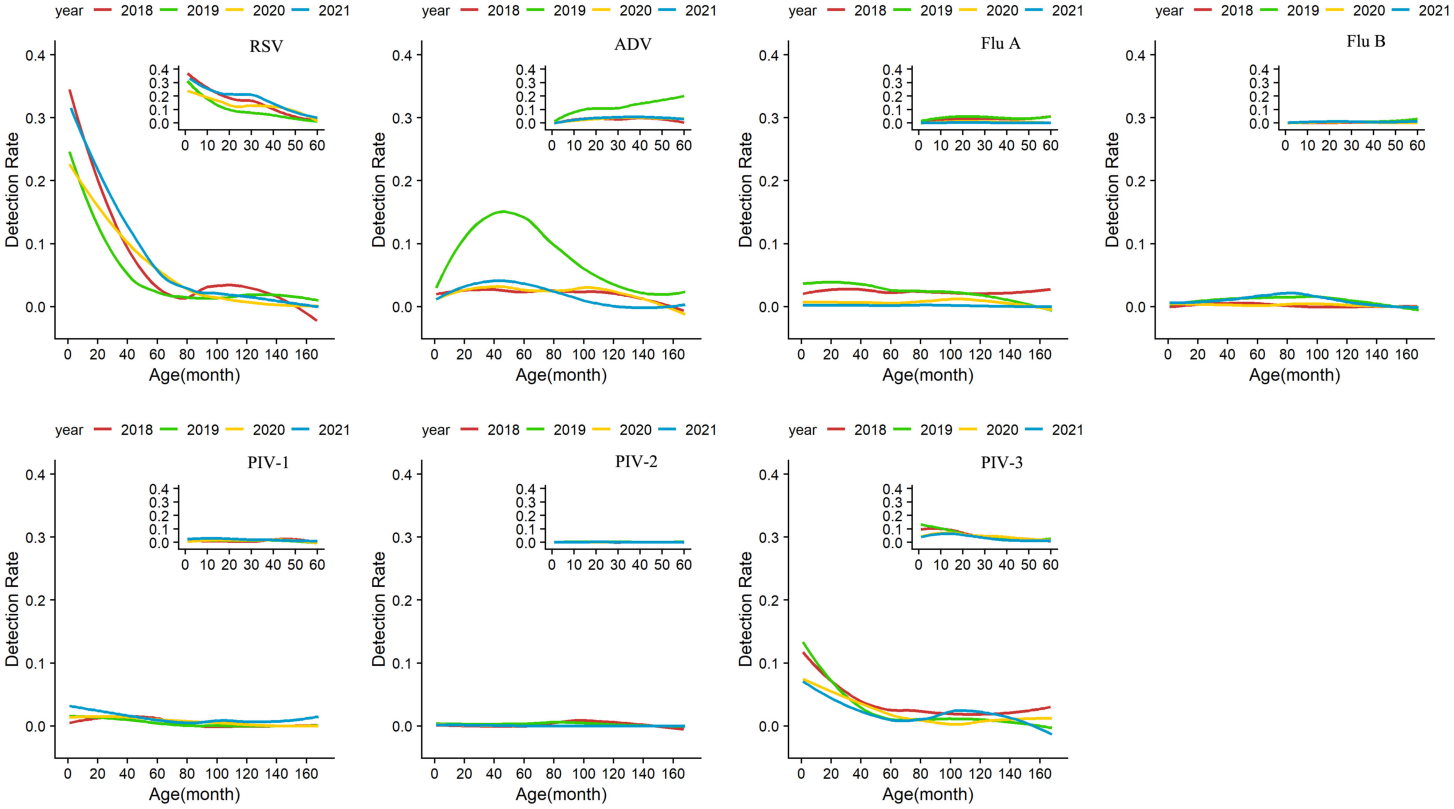
Age-specific detection rates of viruses from January 1, 2018 to December 31, 2021. Seven respiratory viruses were included: respiratory syncytial virus (RSV), adenovirus (ADV), influenza virus A and B (Flu A, Flu B), and parainfluenza virus types 1–3 (PIV1–3).

The overall positive rates reached the peak in children aged 0–12 months, with the peaks of 45.4% in 2018, 42.4% in 2019, 30.5% in 2020, and 38.8% in 2021, and declined with the rising age of the enrolled children. Comparison of age-specific detection rates of seven respiratory viruses in the four years showed that the overall detection rates were greatly reduced among all age groups in 2020 (*P* < 0.001), with most dramatic declines observed in children aged 0–12 months and 1–3 years. The detection rate of Flu A decreased remarkably, and only sporadic cases were detected in all age groups. In 2021, Flu A continued decreasing, while the number of cases with RSV infection among 1–3-year-old children was higher than in the previous three years (*P* < 0.001). Other viruses such as ADV, Flu B, PIV-1, and PIV-3 gradually resumed in 2021 and followed similar patterns as their counterparts in 2018 and 2019 (**Figure 3**; **Supplementary Table**).

## 4 Discussion

Chongqing, located in the southwest of China, swiftly applied the strictest NPIs from January to May 2020 after the outbreak of a novel virus with a high basic reproductive number (R_0_) in Wuhan (Salzberger et al., 2021). Under the circumstances, the total number of children hospitalized for LRTIs in 2020 decreased significantly. The overall detection rate in 2020 was much lower than in 2018 and 2019 (25.3% vs. 37.2% & 34.9%). More specifically, we found that the detection rates of ADV, Flu A, and PIV-3 decreased when NPIs were active from February to August 2020, with Flu A decreasing most predominantly, from 2.7% to 0.3%. Although the overall detection rates of RSV were similar to before, RSV decreased significantly after the outbreak. By continuous surveillance, we also found that the detection rates of RSV and PIV-1 resurged after NPIs lifting and even surpassed the historical level of 2018–2019. Similar results were also described by reports from Italy, South Korea, Hongkong, Shenzhen, Hangzhou, and Shanghai (Liu et al., 2021; Park et al., 2021; Chiu et al., 2022; Nenna et al., 2022; Wang et al., 2022; Ye and Liu, 2022). Multiple factors could contribute to the phenomenon. On the one hand, ADV, Flu A, PIV-1, PIV-3, and RSV are transmitted by contact, droplets, and fomites. Mitigation practices such as wearing a mask, hand disinfecting, physical distancing, home quarantine, and cessation of global travel might have played a protective role in the transmission at the early stage of the pandemic, but have become less stringent in 2021. On the other hand, viral evolution, new viral strains, or mutations might have occurred to circumvent NPIs for the COVID-19 pandemic.

Significant decreases in viral infection in all age groups were reported in 2020 compared with the previous two years, which was particularly notable in children aged 0–12 months and 1–3 years. This may be due to the lower exposure to viruses caused by various NPIs in Chongqing. For example, employees worked at home and held meetings online, and therefore children had less contact with anyone other than household members during the COVID-19 pandemic. Our results might help illustrate the role of household transmission of respiratory viruses from older siblings and adults.

RSV is the most important viral pathogen causing acute LRTIs in children under the age of 5 years (Shi et al., 2017). In this study, RSV was the most frequently detected virus among children younger than 6 years old with LRTIs. The detection rate of RSV sharply dropped after an outbreak in January 2020 and stayed in a nearly dormant state during the next seven months. However, the detection rates from June to September of 2021 remained abnormally high (over 10%), as reported in Shanghai and Beijing (Jia et al., 2022; Jiang et al., 2022). We also found higher proportions of RSV-infected children aged 1–3 years in 2021. Multinational studies have also reported atypical seasonal characteristics of RSV during the pandemic (Casalegno et al., 2021; Ohnishi and Kawano, 2021; Weinberger Opek et al., 2021; Mohebi et al., 2022). In a recent study from Australia, researchers reported up to a 98% reduction in RSV cases and unexpected outbreaks since September 2020 as physical restrictions relaxed, with increased age distribution (median age, 18.4 months vs. 7.3–12.5 months in 2012–2019) (Foley et al., 2021). There was a rapid increase in RSV cases in New Zealand, and the surveillance numbers after a partial relaxation of the strict border closure policy in April 2021 were five times higher than the 2015–2019 peak average. It was also shown that the number of infected children aged 0–4 years was three times higher than the peak average in 2015–2019 (Hatter et al., 2021). South Korea reported an outbreak of RSV in November 2021 after more than 1-year zero detection, when public measures were lifted and the SARS-Cov-2 vaccination was implemented. However, no differences in median age were reported (Kim et al., 2022). Factors contributing to this usual summer peak are complicated, and relaxation of NPIs has likely played a predominant role. Moreover, this out-of-season upsurge might also be related to the emergence of new viral genetic lineage strains. Original research conducted in Taiwan, a province of China, reported that the delay of RSV outbreak in 2020 was caused by two novel RSV ON1.1 variants (Lee et al., 2022). A study in Argentina also showed that the delayed RSV upsurge was related to new viral variants (Dolores et al., 2022). Epidemiological data in Beijing revealed abnormal RSV prevalence in the summer of 2021 along with the shift of subtypes from B to A (Jiang et al., 2022).

The influenza virus is another important pathogen for LRTIs and has caused severe disease burdens worldwide (Li et al., 2022). However, we observed almost no Flu A and Flu B since the outbreak in late January 2020. Low detection of influenza viruses persisted even after full school reopening, work resuming, and distance shortening in 2021. A model predicted that future influenza viruses rebound would occur after the resumption of social activities, due to the low exposure and an overall increase of susceptibility in society during the pandemic (Baker et al., 2020). However, a lower-than-usual magnitude was also observed in the winter of 2021. A multicenter study showed that the 2020–2021 seasonal influenza epidemics were delayed, shorter, and less intense, and that school closings, canceling of public events, and restricting internal movements seemed to reduce transmissions (Davis et al., 2022). NPIs applied during COVID-19 may have played a vital role in the process. Furthermore, negative interactions between Flu A and rhinovirus (RV) may have also played a role (Piret and Boivin, 2022). It has been reported that RV increased sharply after the reopening of schools in 2020, exceeding the same period of previous years (Poole et al., 2020; Redlberger-Fritz et al., 2021; Takashita et al., 2021). However, we did not analyze epidemiological information of RV in this report. It is necessary to continue monitoring RV and influenza viruses in our following project. Influenza virus vaccination was not a convincing factor. Under the impact of the COVID-19 pandemic, the willingness to receive influenza vaccines has been increasing to avoid double infections of SARS-CoV-2 and influenza virus (Hou et al., 2021). Even so, the influenza virus vaccination coverage in China, 9.4% among the general population (Cohen et al., 2021), was very low compared with other countries and far from the herd immunity threshold (Fine et al., 2011).

Previous studies have shown that PIV-3 circulates most frequently from late spring to summer, and its detection rate in winter is low (Li et al., 2019). However, there was a very low prevalence from January to July 2020, but a sharp increase from August to November, when schools reopened and NPIs relaxation started, with a delay of about 5 months. Out-of-season trends of PIV-3 were reported in other studies as well (Liu et al., 2021; Takashita et al., 2021; Kim et al., 2022; Ye and Liu, 2022). However, the mechanism behind this atypical pattern is not yet clear. Lifted travel restrictions, reopening of educational institutions, and resumption of socioeconomic activities may all have triggered this unusual epidemic. These results also indicate that the healthcare system may need to prepare for future atypical prevalence of common respiratory viruses such as influenza viruses.

This study has several limitations. First, this was a single-center study, and we only collected data on patients with LRTIs, who were more likely to be severely affected, with a selection bias. Multicenter research trials and the inclusion of larger patient cohorts in the future will provide help for the control, prevention, and treatment of respiratory virus infections. Second, this was a retrospective analysis that only surveyed a small proportion of viral respiratory antigens by direct immunofluorescence assay; thus, further epidemiological surveillance of more pathogens by molecular assay and their circulating subtypes will help reveal the more-detailed epidemiological information. Third, this study was conducted in 2021, when strict NPIs were continued in Chongqing with varying stringency according to the number of local and imported cases of SARS-CoV-2 infections, and we did not observe the large-scale out-of-season outbreaks as reported abroad. More multicenter epidemiological studies are needed when mitigation measures are completely lifted.

## 5 Conclusion

In this study, we enrolled more than 30,000 hospitalized children diagnosed with LRTIs with a time span covering two years before and during the epidemic to explore the prevalence of the respiratory viruses. We summarized that the NPIs implemented during the COVID-19 pandemic had great impacts on the prevalence of common respiratory viruses and seasonal patterns of certain viruses such as RSV, PIV-3, and influenza viruses. Clinicians should be aware that seasonal respiratory viruses might not exhibit typical circulation patterns and be cautious of a possible unusual rebound and unexpected resurgence of seasonal respiratory viruses, such as influenza viruses, when sanitary restrictions are fully lifted. Therefore, we suggest continuous surveillance of the epidemiological and evolutionary dynamics of multiple respiratory pathogens, especially when major public policies are launched.

## Data availability statement

The raw data supporting the conclusions of this article will be made available by the authors, without undue reservation.

## Ethics statement

The study protocol was approved by the Ethics Committee of Children’s Hospital of Chongqing Medical University (File No. (2022)196). Written consent was waived because antigen detection of respiratory pathogens is standard and routine for all children admitted for LRTIs in the hospital while the study was implemented.

## Author contributions

DT designed the project. Material preparation and data collection were performed by LZ, TL, and XW. Data was analyzed by SY, YY, TL, and LZ. This program was guided by ZF, EL, DT, JL, and XX. Manuscript drafted by LZ. TG, and CN revised the manuscript. All authors contributed to the article and approved the submitted version.
