# Supplementary material for: Epidemiological characteristics of respiratory viruses in hospitalized children during the COVID-19 pandemic in southwestern China
Source: Front Cell Infect Microbiol. 2023 Apr 4;13:1142199. doi: 10.3389/fcimb.2023.1142199 (PMC10157792; doi:10.3389/fcimb.2023.1142199)
Supplement: Supplementary file 1 [file DataSheet_1.doc]

Supplementary Materials for final proof

# Supplementary Table and Figure

**Supplementary Table** Detection rates of respiratory virus infection based on age from 2018-2021

| **Year, Virus, Age** | | 2018, n=8141 | 2019, n=8681 | 2020, n=6252 | 2021, n=8059 | **χ^2^** | P |
| --- | --- | --- | --- | --- | --- | --- | --- |
| RSV | 0-12m | 1563(32.0) | 1010(23.7) | 772(23.0) | 1071(29.3) | 119.831 | <0.001 |
|  | 1-3y | 357(18.7) | 223(10.3) | 207(13.7) | 434(20.8) | 105.848 | <0.001 |
|  | 3-6y | 49(5.5) | 54(3.6) | 73(8.6) | 145(8.7) | 41.575 | <0.001 |
|  | 6-14y | 12(2.7) | 14(2.0) | 8(1.6) | 10(1.6) | 2.095 | 0.553 |
| ADV | 0-12m | 65(3.4) | 223(5.2) | 31(0.9) | 47(1.3) | 23.701 | <0.001 |
|  | 1-3y | 64(3.4) | 246(11.3) | 50(3.3) | 77(3.7) | 179.552 | <0.001 |
|  | 3-6y | 30(3.4) | 242(16.1) | 26(3.1) | 66(4.0) | 238.501 | <0.001 |
|  | 6-14y | 11(2.5) | 47(6.6) | 9(1.8) | 8(1.3) | 36.451 | <0.001 |
| Flu A | 0-12m | 88(1.8) | 115(2.7) | 10(0.3) | 5(0.1) | 134.039 | <0.001 |
|  | 1-3y | 59(3.1) | 106(4.9) | 14(0.9) | 5(0.3) | 116.799 | <0.001 |
|  | 3-6y | 24(2.7) | 43(2.9) | 7(0.8) | 3(0.2) | 47.429 | <0.001 |
|  | 6-14y | 9(2.0) | 15(2.1) | 3(0.6) | 1(0.2) | 14.314 | 0.003 |
| Flu B | 0-12m | 9(0.2) | 20(0.5) | 4(0.1) | 20(0.5) | 15.444 | 0.001 |
|  | 1-3y | 4(0.2) | 19(0.9) | 10(0.7) | 19(0.9) | 9.152 | 0.027 |
|  | 3-6y | 4(0.4) | 20(1.3) | 1(0.1) | 15(0.9) | 11.663 | 0.009 |
|  | 6-14y | 0(0.0) | 8(1.1) | 2(0.4) | 12(1.9) | 12.470 | 0.006 |
| PIV-1 | 0-12m | 48(1.0) | 56(1.3) | 34(1.0) | 116(3.2) | 75.590 | <0.001 |
|  | 1-3y | 17(0.9) | 33(1.5) | 27(1.8) | 49(2.3) | 13.493 | 0.004 |
|  | 3-6y | 15(1.7) | 14(0.9) | 14(1.6) | 25(1.5) | 4.504 | 0.212 |
|  | 6-14y | 0(0.0) | 1(0.1) | 2(0.4) | 4(0.6) | 3.093 | 0.378 |
| PIV-2 | 0-12m | 3(0.1) | 11(0.3) | 4(0.1) | 3(0.1) | 7.833 | 0.050 |
|  | 1-3y | 0(0.0) | 8(0.4) | 3(0.2) | 4(0.2) | 7.070 | 0.070 |
|  | 3-6y | 1(0.2) | 4(0.3) | 0(0.0) | 0(0.0) | 6.556 | 0.087 |
|  | 6-14y | 1(0.2) | 2(0.3) | 0(0.0) | 0(0.0) | 2.977 | 0.395 |
| PIV-3 | 0-12m | 527(10.8) | 538(12.6) | 203(6.1) | 227(6.2) | 152.821 | <0.001 |
|  | 1-3y | 130(6.8) | 129(5.9) | 92(6.1) | 98(4.7) | 8.461 | 0.037 |
|  | 3-6y | 28(3.2) | 25(1.7) | 25(2.9) | 24(1.4) | 12.730 | 0.005 |
|  | 6-14y | 10(2.3) | 9(1.3) | 5(1.0) | 8(1.3) | 3.173 | 0.366 |
| Overall | 0-12m | 2219(45.4) | 1851(42.4) | 1024(30.5) | 1424(38.3) | 205.596 | <0.001 |
|  | 1-3y | 620(32.5) | 700(32.2) | 384(25.3) | 652(31.2) | 25.810 | <0.001 |
|  | 3-6y | 146(16.4) | 385(25.6) | 140(16.5) | 267(16.0) | 59.226 | <0.001 |
|  | 6-14y | 41(9.3) | 95(13.3) | 29(5.7) | 43(7.0) | 25.496 | <0.001 |

Annual detection rate of children aged >14y were not compared and showed in order to reduce errors caused by small sample sizes.

**
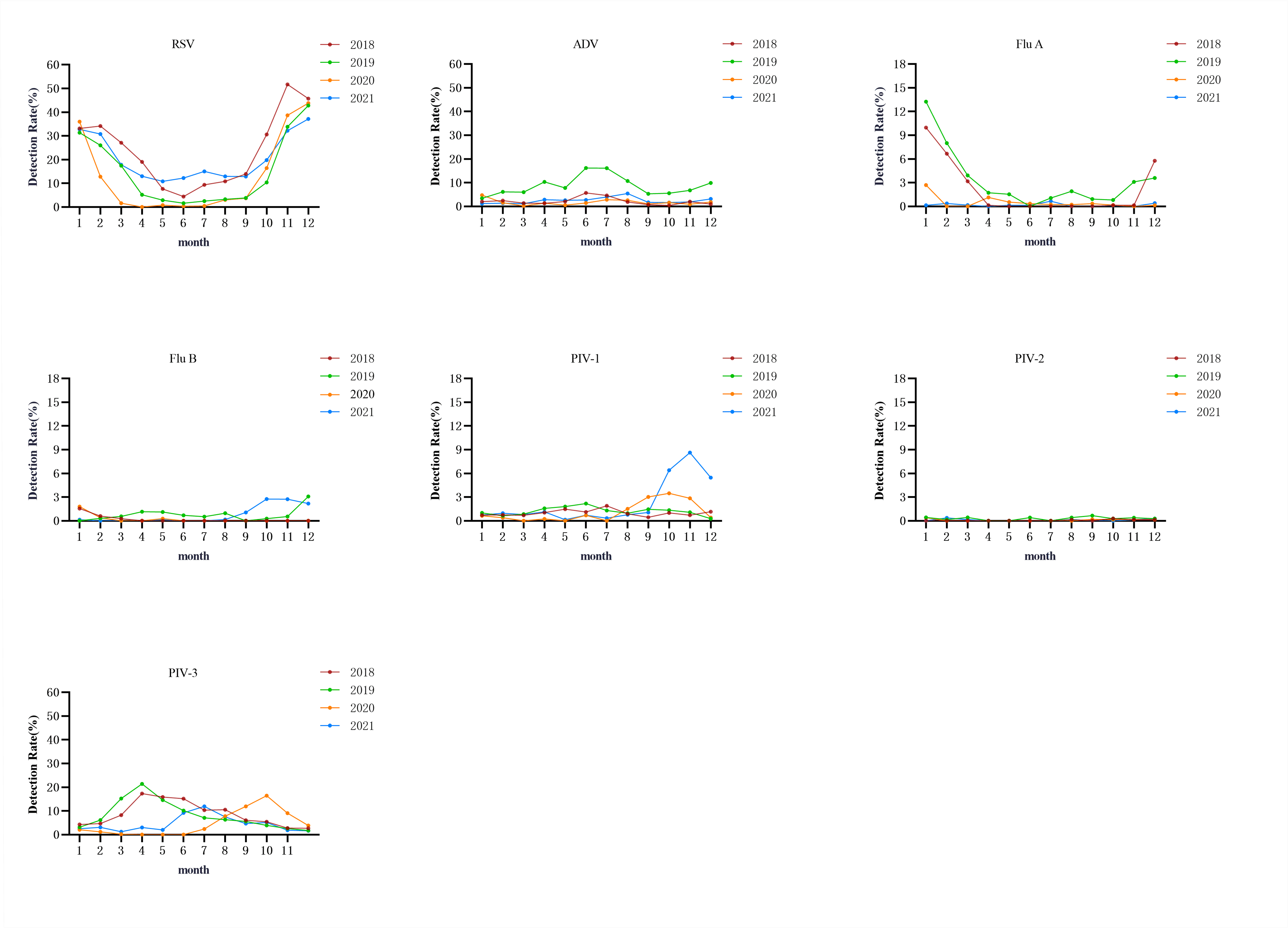
**

**Supplementary Figure**. Monthly detection rates of respiratory syncytial virus (RSV), adenovirus (ADV), influenza A (Flu A), influenza B (Flu B) and parainfluenza virus (PIV)1-3 from 2018-2021.
